# A Laboratory Guide in Urinalysis and Toxicology

**Published:** 1899-06

**Authors:** 


A Laboratory Guide in Urinalysis and Toxicology. By R. A. Witthaus, A. M., M. D., Professor of Chemistry, Physics and Toxicology in the Medical Department of Cornell University, etc. Fourth edition. New York. William Wood & Co. 1898. Price, $1.00 net.

This little book is an outline of urinalysis and toxicology as taught by Prof. Witthaus, and is intended primarily for his students. The subject matter is admirably arranged as a result of long experience in teaching, and, which serving as the best guide in Cornell and the Medical Department of the University of Vermont, institutions in which Professor Witthaus is the teacher of Chemistry, the guide is suitable for use in other institutions and seems to be the favorite in many of them. The book has 111 pages, with alternating blank pages for notes. Doremus' ureometer is not mentioned in the book. T. J. B.

